# Apalutamide Sensitizes Prostate Cancer to Ionizing Radiation via Inhibition of Non-Homologous End-Joining DNA Repair

**DOI:** 10.3390/cancers11101593

**Published:** 2019-10-18

**Authors:** Wenhao Zhang, Chen-Yi Liao, Hajar Chtatou, Luca Incrocci, Dik C. van Gent, Wytske M. van Weerden, Julie Nonnekens

**Affiliations:** 1 Department of Molecular Genetics, Erasmus MC, PO box 2040, 3000 CA Rotterdam, The Netherlands; w.zhang@erasmusmc.nl (W.Z.); d.vangent@erasmusmc.nl (D.C.v.G.); 2 Department of Experimental Urology, Erasmus MC, PO box 2040, 3000 CA Rotterdam, The Netherlands; c.liao@umail.leidenuniv.nl (C.-Y.L.); Hajarchtatou24@icloud.com (H.C.); w.vanweerden@erasmusmc.nl (W.M.v.W.); 3 Department of Radiation Oncology, Erasmus MC Cancer Institute, PO box 2040, 3000 CA Rotterdam, The Netherlands; l.incrocci@erasmusmc.nl; 4 Oncode Institute, Erasmus MC, PO box 2040, 3000 CA Rotterdam, The Netherlands; 5 Department of Radiology and Nuclear Medicine, Erasmus MC, PO box 2040, 3000 CA Rotterdam, The Netherlands

**Keywords:** prostate cancer, radiosensitization, external beam radiation therapy, apalutamide, anti-androgens, non-homologous end-joining

## Abstract

Androgen-deprivation therapy was shown to improve treatment outcome of external beam radiation therapy (EBRT) for locally advanced prostate cancer (PCa). DNA damage response (DDR) was suggested to play a role in the underlying mechanism, but conflicting results were reported. This study aims to reveal the role of the androgen receptor (AR) in EBRT-induced DDR and to investigate whether next-generation AR inhibitor apalutamide can radiosensitize PCa. PCa cell lines and tissue slices were treated with anti-androgen alone or combined with EBRT. The effect of treatments on cell growth, tissue viability, DDR, and cell cycle were investigated. RAD51 and DNA-dependent protein kinase catalytic subunit (DNA-PKcs) levels were determined by Western blotting. Homologous recombination (HR) capacity was measured with the directed repeats-green fluorescent protein (DR-GFP) assay. We report the radiosensitizing effect of anti-androgens, which showed synergism in combination with EBRT in AR-expressing tumor slices and cell lines. Moreover, a compromised DDR was observed in AR-expressing cells upon AR suppression. We found that AR inhibition downregulated DNA-PKcs expression, resulting in reduced non-homologous end-joining repair. DDR through HR was a secondary effect due to cell-cycle change. These data provide a mechanistic explanation for the combination regimen and support the clinical use of apalutamide together with EBRT for localized PCa patients.

## 1. Introduction

Prostate cancer (PCa) is the most common malignancy and the second most common cause of cancer-related death among men [1]. External beam radiation therapy (EBRT) is the first line of treatment for locally advanced PCa; however, unfortunately, tumors frequently recur after treatment [2,3]. The combination of EBRT with androgen-deprivation therapy (ADT), the first line of systemic treatment for advanced PCa, was shown to improve the overall therapeutic outcome compared to EBRT alone [4,5,6]. Blocking activation of the androgen receptor (AR) signaling pathway by ADT is highly effective in reducing PCa tumor growth; however, preclinical studies and clinical trials were not able to assess whether the addition of ADT to EBRT induces a synergistic or additive effect. Moreover, conflicting results were reported on the underlying cellular mechanisms causing this effect. Interestingly, recent studies indicated that AR signaling can activate DNA repair pathways, which may contribute to radioresistance [7,8].

Ionizing radiation (IR) induces DNA double-strand breaks (DSBs), which can be repaired via two main pathways: Non-homologous end-joining (NHEJ) and homologous recombination (HR). NHEJ involves recruitment of DNA-dependent protein kinase catalytic subunit (DNA-PKcs) to DNA ends by the Ku70/80 heterodimer, followed by coupling of the DNA ends. HR is mediated by the RAD51 protein in combination with many other factors such as breast cancer 1 and 2 (BRCA1 or 2) [9]. AR activation promotes upregulation of DNA-PKcs, Ku70/80, and RAD51; however, it is unclear whether AR directly regulates expression of these genes, and previous studies contained conflicting findings on DNA repair pathway regulation [7,8,10,11].

Apalutamide (ERLEADA^®^) is a next-generation AR inhibitor which was approved in 2018 for treatment of non-metastatic castration-resistant prostate cancer (CRPC). Apalutamide blocks AR activation by competing with androgen binding and preventing AR translocation to the nucleus. It has a 5–10-fold higher binding affinity than the first-generation anti-androgen bicalutamide, which is widely used for PCa patient treatment [12]. Also, apalutamide showed a greater efficacy compared to the second-generation anti-androgen enzalutamide [12], which was shown in preclinical studies to have radiosensitizing capacities [13]. Ongoing clinical trials are testing the efficacy of combination treatment of apalutamide and EBRT; however, preclinical data are yet to be presented to support this combination.

Here, we compared the radiosensitizing capacity of apalutamide to ADT and enzalutamide in PCa cells and ex vivo patient-derived xenograft (PDX) tissue slice models. Furthermore, we identified NHEJ downregulation as the underlying mechanism of apalutamide-enhanced radiosensitivity in AR-expressing PCa models.

## 2. Results

### 2.1. AR Suppression Enhances IR-Induced Cell Killing

**Figure 1:**
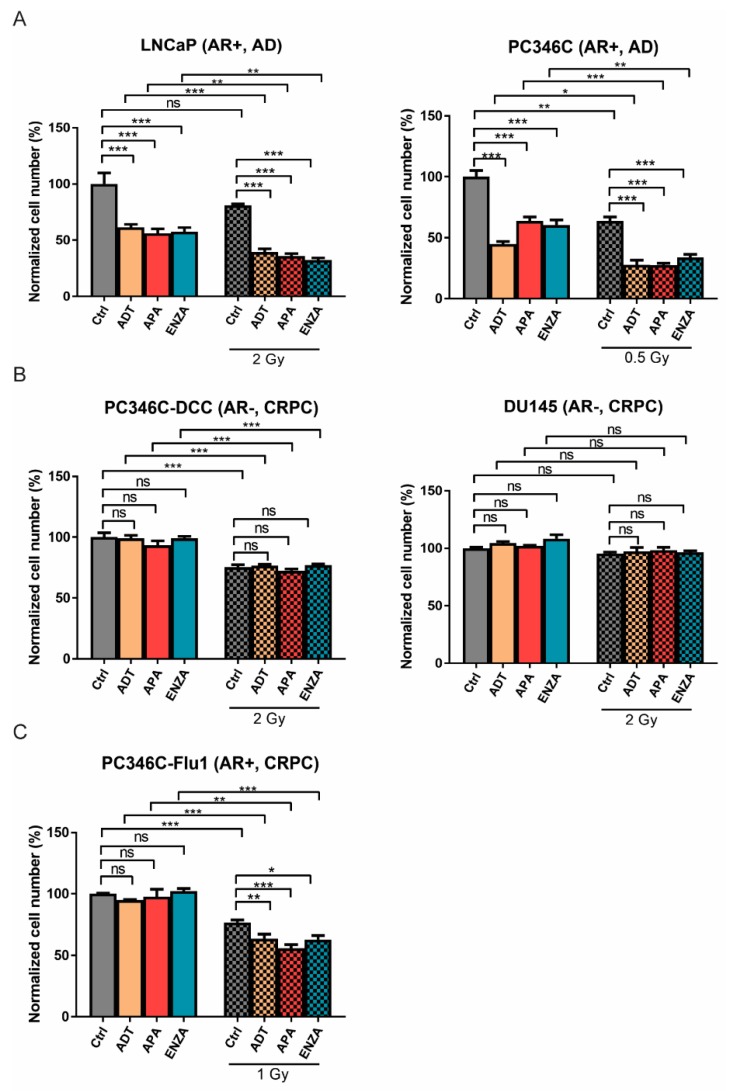
Androgen receptor (AR) suppression treatment inhibits cell growth cooperatively with ionizing radiation (IR) treatment in AR-positive prostate cancer (PCa) cells. Cells were pretreated with AR suppression or dimethyl sulfoxide (DMSO) control for 24 h followed by IR treatment. The cell number was measured at day seven post-treatment with a sulforhodamine beta (SRB) assay and set relative to initial cell number followed by normalized to untreated control. (**A**) Androgen receptor (AR)-positive and androgen-dependent (AD) LNCaP and PC346C; (**B**) AR-negative, castration-resistant prostate cancer (CRPC) PC346C-DCC and DU145 cells; (**C**) AR-positive and CRPC PC346C-Flu1 cells. Averages and standard errors of the mean (SEM) are indicated; * *p* < 0.05, ** *p* < 0.01, *** *p* < 0.001, ns, non-significant, ADT, androgen-deprivation treatment; APA, apalutamide; ENZA, enzalutamide.

The impact of AR suppression treatments on PCa cell response to IR exposure was studied by applying ADT, enzalutamide, or apalutamide 24 h prior to IR. The IR dose per cell lines was chosen such that cell proliferation was not fully inhibited. Since the intrinisic radiosensitivity varied between the tested cell lines, a direct comparison of the observed radiosensitizing effects between these cell lines was difficult. AR suppression alone reduced the total cell number by 50% in LNCaP and 40% in PC346C cells seven days post-IR, and IR treatment alone reduced the total cell number by 20% in LNCaP and 35% in PC346C cells (Figure 1A). The combination treatment resulted in a further reduction of the total cell number by 65% and 70% for LNCaP and PC346C cells, respectively. As expected, AR suppression did not affect cell number in AR-negative DU145 and PC346C-DCC cells, and the addition of AR suppression to IR exposure did not cause additional sensitivity (Figure 1B). In AR-overexpressing PC346C-Flu1 cells, AR suppression did not affect cell growth, reflecting the CRPC nature of this cell line. Interestingly, combining AR suppression and IR treatments resulted in a small, but significant reduction of the total cell number with 40% at seven days post-IR compared to IR alone (20%) (Figure 1C). No significant difference was observed between the different AR suppression treatments. These data suggest that there is an additional effect of the combination treatment compared to IR alone, which is dependent on the AR status of the tumor cells.

### 2.2. Apalutamide Enhances PCa Tissue Slice IR Sensitivity

**Figure 2:**
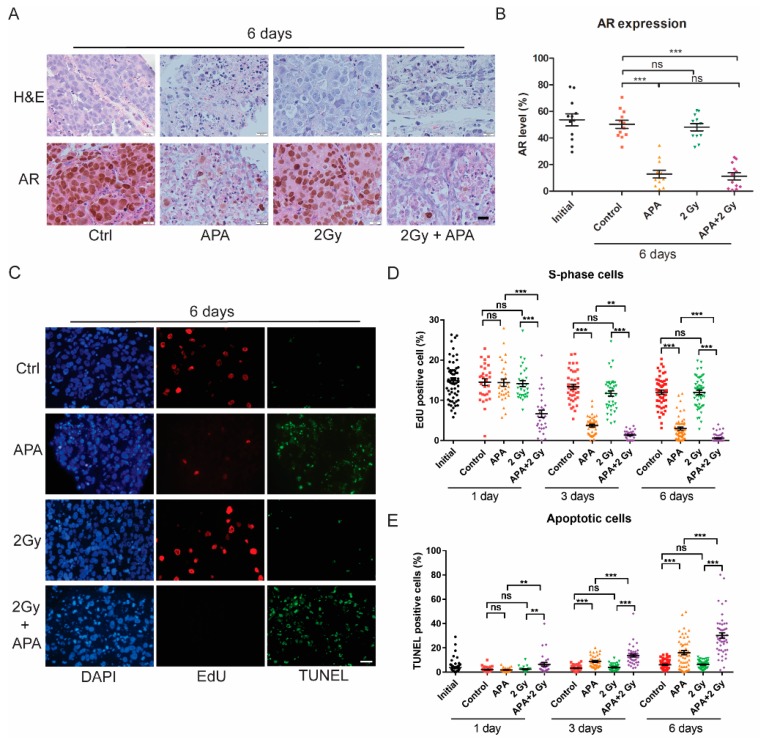
Apalutamide radiosensitizes PCa tissue slices ex vivo. Androgen receptor (AR)-positive, androgen-dependent PC295 patient-derived xenograft (PDX) slices were pretreated with apalutamide (APA) for 2 h followed by 2 Gy of IR treatment. (**A**) Representative hematoxylin and eosin (H&E) and AR immunostaining images of tumor slice sections after six days of culturing under different conditions; scale bar = 20 μm. (**B**) Quantification of AR expression. Four image fields were analyzed per tumor slice, where each data point represents one image field; averages and standard error of the mean (SEM) are indicated. (**C**) Representative 4′,6-diamidino-2-phenylindole (DAPI), 5-ethynyl-2’-deoxyuridine (EdU), Terminal deoxynucleotidyl transferase dUTP nick end labeling (TUNEL) images of tumor slice sections after six days of culturing under different conditions; scale bar = 20 μm. (**D**) Quantification of the fraction of EdU-positive cells in the tissue slices. (**E**) Quantification of the fraction of TUNEL-positive cells in the tissue slices. For EdU and TUNEL graphs, 10 image fields were analyzed per tumor slice. Each data point represents one image field; averages and SEM are indicated; ** *p* < 0.01, *** *p* < 0.001, ns, non-significant.

Subsequently, we investigated the effects of apalutamide on IR sensitivity in androgen-dependent, AR-positive PC295 ex vivo tumor slices [14]. Tumor slices retained morphology six days post IR-exposure, while treatment with apalutamide resulted in tissue integrity loss and loss of AR expression (Figure 2A,B), and significantly reduced tissue slice viability with a reduced fraction of synthesis (S)-phase cells and increased number of apoptotic cells (Figure 2C–E). Furthermore, 2 Gy of IR had no effect on tissue slice viability, with neither a reduction of S-phase cells nor an increase of apoptotic cells at any time point. Combination of IR and apalutamide significantly reduced the level of S-phase cells and increased the level of apoptotic cells at day one after IR, at which no effect of the single treatments could be detected, indicating a synergy between these two treatments. This effect became more prominent over time (Figure 2C–E). The combination treatment did not further reduce AR expression level compared to apalutamide treatment alone (Figure 2A,B). Together, these data show the synergistic effect of IR and apalutamide treatment in this PCa tumor model.

### 2.3. AR Suppression Inhibits DNA Damage Repair

**Figure 3:**
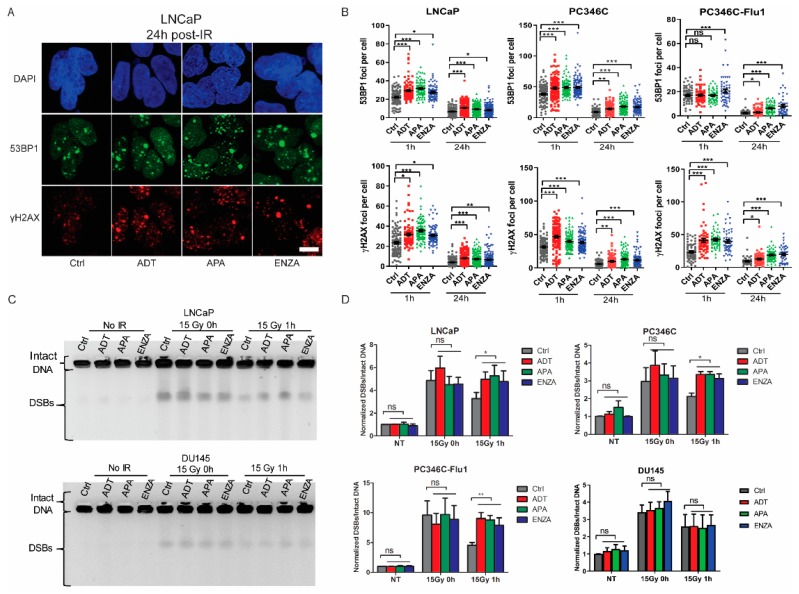
AR suppression treatment suppresses DNA damage repair. (**A**) Cells were treated with AR suppression for 24 h followed by 2 Gy of IR treatment. Representative images of phospho-H2A histone family member X (γ-H2AX) and p53-binding protein 1 (53BP1) staining 24 h post-IR of LNCaP cells (scale bar 10 μm). (**B**) Quantification of γH2AX and 53BP1 foci from different cell lines. Each data point represents one cell; averages and SEM are indicated. (**C**) Representative pulsed-field gel electrophoresis (PFGE) image of PC346C and DU145 cells under different conditions. (**D**) DNA break signals were quantified using ImageJ and normalized to unsaturated signals of intact DNA. Averages and standard deviations are indicated; * *p* < 0.05, ** *p* < 0.01, *** *p* < 0.001, ns, non-significant. ADT, androgen-deprivation treatment; APA, apalutamide; ENZA, enzalutamide.

**Figure A1:**
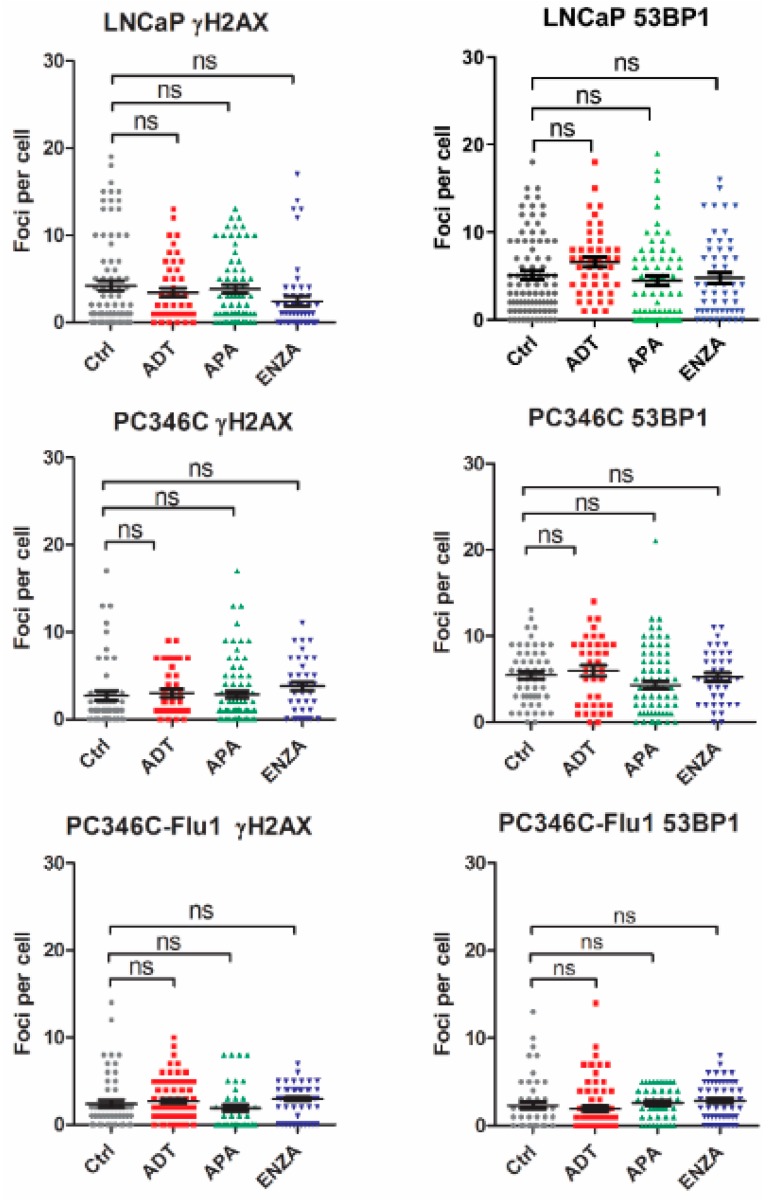
Quantification of γH2AX and 53BP1 foci in cell lines treated with AR suppression for 48 h. Each data point represents one cell; averages and SEM are indicated; ns, non-significant. ADT, androgen-deprivation treatment; APA, apalutamide; ENZA, enzalutamide.

**Figure A2:**
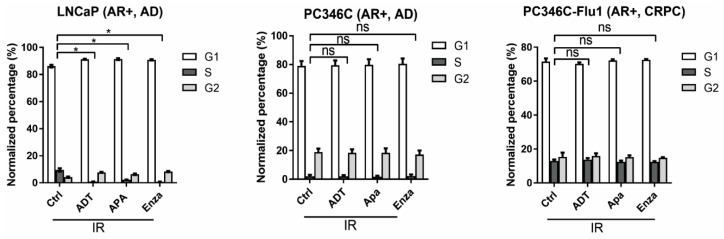
LNCaP, PC346C, and PC346C-Flu1 cells were treated with AR suppression for 24 h followed by IR (2 Gy). Cell samples were collected 24 h post-IR, and cell cycle was determined by flow cytometry. Averages and SD are indicated; * *p* < 0.05, ns, non-significant. ADT, androgen-deprivation treatment; APA, apalutamide; ENZA, enzalutamide.

Next, we investigated whether AR suppression treatment-enhanced IR sensitivity could result from a DSB repair defect by measuring DSB repair kinetics of PCa cells. AR suppression treatment alone did not increase DSB foci numbers compared to untreated controls (Figure A1, Appendix). IR exposure resulted in rapid induction of DSBs, measured by phospho-H2A histone family member X (γ-H2AX) and p53-binding protein 1 (53BP1) foci staining, in all three cell lines tested (Figure 3A,B). At 1 and 24 h after IR, cells in the combination therapy groups showed significantly more DSB foci than cells treated with IR alone, indicative of a slower repair of DSBs or a higher level of DNA damage (Figure 3A,B). An alternative explanation for the increase of foci numbers could be the increased DNA content per nucleus in the S/gap 2 (S/G2) phase of the cell cycle. Therefore, we analyzed the cell-cycle distribution 24 h post-IR. The fraction of S/G2 cells was not increased by AR suppression treatment in PC346C and PC346C-Flu1 cells (Figure A2, Appendix), while LNCaP cells displayed an even higher fraction of gap 1 (G1) phase cells, a cell phase with less DNA content, excluding a cell-cycle effect.

In addition to indirect measurement of the level of DSBs using phospho-H2A histone family member X (γH2AX) and p53-binding protein 1 (53BP1) foci quantifications, we also measured the number of DNA breaks by pulsed-field gel electrophoresis (PFGE). AR suppression as single treatment did not cause increased levels of broken DNA, while IR exposure did. No difference was observed between control and AR suppression treatment directly after IR exposure, indicating that the same level of DNA breaks was induced (Figure 3C,D). When allowing cells to repair the DNA damage for 1 h, a significantly higher level of DNA breaks was observed in AR suppression treated cells compared with the control group (Figure 3C,D). This effect was robust in both androgen-dependent and CRPC AR-expressing cells, but was absent in AR-negative DU145 cells (Figure 3C,D). Combined, these data indicate that AR suppression inhibits repair of IR-induced DSBs and that this effect is dependent on AR expression.

### 2.4. Reduced HR Efficiency Provoked by AR Suppression Results from Cell-Cycle Alterations

**Figure 4:**
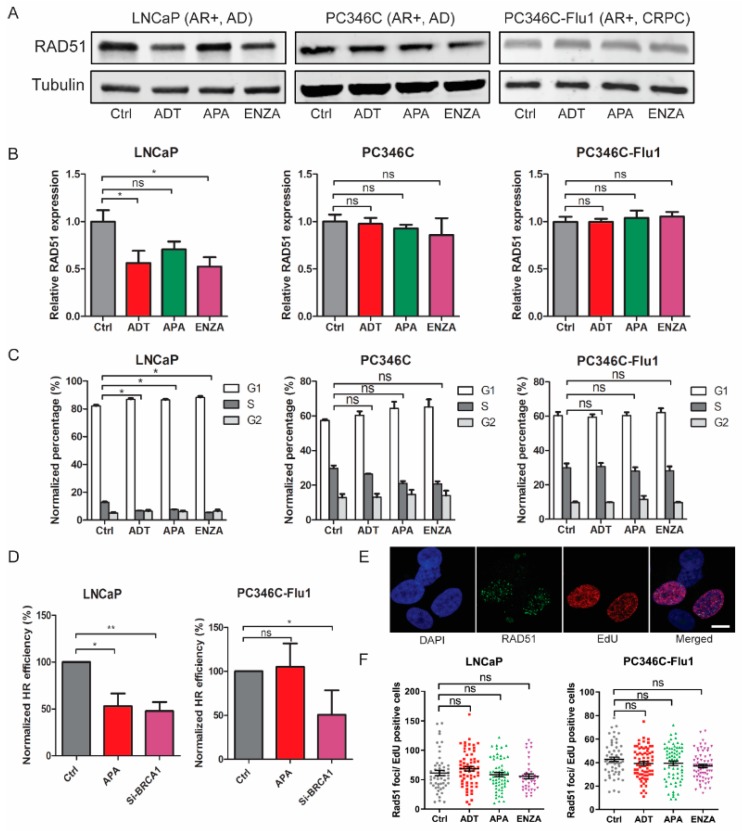
Reduced homologous recombination (HR) efficiency provoked by AR suppression treatment results from cell-cycle alterations. (**A**) LNCaP, PC346C, and PC346C-Flu1 cells were treated with AR suppression for 48 h and RAD51 expression was analyzed by Western blotting. Tubulin was used as a loading control. Full blots can be found in Figure A3 (Appendix). (**B**) Quantification of RAD51 protein levels compared to loading control and normalized to untreated (Ctrl). Averages and SEM are indicated. (**C**) The same cell samples from a were incubated with EdU for 30 min before fixation and the cell-cycle profile was determined by flow cytometry. Averages and SD are indicated. (**D**) A transient directed repeats-green fluorescent protein (DR-GFP) assay was performed in LNCaP and PC346C-Flu1 cells treated with apalutamide for 48 h. Cells transfected with small interfering RNA (siRNA) against breast cancer 1 (BRCA1) transcript for 72 h were used as a positive control. GFP-positive cells were scored by flow cytometry and quantified. Averages and SD are indicated. (**E**) Representative images of double staining of EdU-positive and RAD51 foci and their colocalization in LNCaP cells at 2 h after 5 Gy of IR treatment (scale bar = 10 μm). (**F**) Quantification of RAD51 foci numbers in EdU-positive cells. Each data point represents one cell; averages and SEM are indicated; * *p* < 0.05, ** *p* < 0.01, ns, non-significant. ADT, androgen-deprivation treatment; APA, apalutamide; ENZA, enzalutamide.

Subsequently, we investigated whether AR suppression directly affected HR. Since previous studies reported reduced RAD51 expression after enzalutamide treatment [10], we measured changes in the RAD51 protein level after the different AR suppression treatments. In LNCaP cells, a reduction of RAD51 expression was detected upon AR suppression (statistically significant after ADT and enzalutamide treatment) (Figure 4A,B). However, PC346C and PC346C-Flu1 cells did not show similar decrease in RAD51 levels after AR suppression treatment (Figure 4A,B). Cell-cycle analysis revealed a significant reduction of the fraction of S-phase cells in LNCaP cells, while no change was observed in PC346C and PC346C-Flu1 cells (Figure 4C). Since RAD51 is only expressed in S/G2 phase cells, this suggests that reduced RAD51 levels may be secondary to cell-cycle distribution changes. To further substantiate this finding, the effect of apalutamide treatment on HR was directly assessed using the directed repeats-green fluorescent protein (DR-GFP) assay. Apalutamide treatment caused a 50% reduction of HR in LNCaP cells (Figure 4D), while PC346C-Flu1 cells were not affected. As a control, knockdown of the BRCA1 protein successfully reduced HR capacity more than 50% in both cell lines (Figure 4D). Since HR functionality was reduced upon apalutamide treatment only in LNCaP cells, these data suggest again that this may be caused by a reduced fraction of S/G2 cells (Figure 4C). To confirm that HR capacity was not affected in S/G2 cells, we measured HR functionality by RAD51 foci formation in EdU-positive cells. RAD51 foci formation in S-phase cells was not changed by AR suppression in both LNCaP and PC346C-Flu1 cells (Figure 4E,F). Taken together, these data indicate that HR efficiency is not directly inhibited by AR suppression, but is only a secondary effect of cell-cycle alteration.

### 2.5. NHEJ Inhibition by AR Suppression Contributed to Radiosensitization

**Figure 5:**
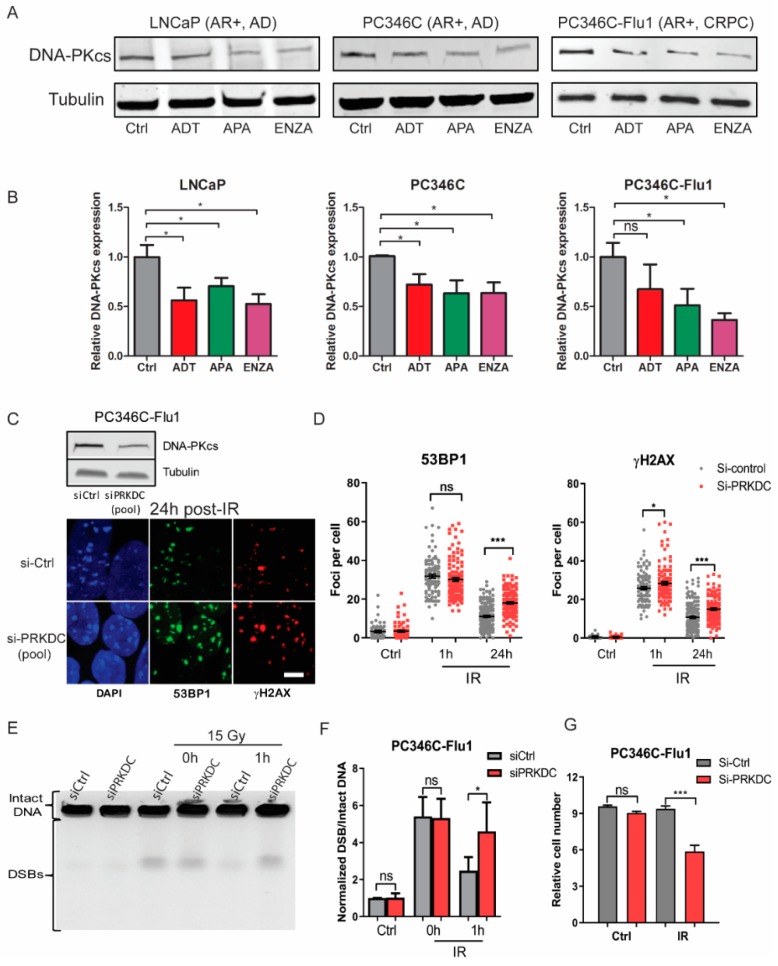
Non-homologous end-joining (NHEJ) suppression is regulated by AR signaling and contributes to radiosensitization. (**A**) LNCaP, PC346C, and PC346C-Flu1 cells were treated with AR suppression for 48 h, and DNA-dependent protein kinase catalytic subunit (DNA-PKcs) protein levels were analyzed by Western blotting. Tubulin was used as a loading control. Full blots can be found in Figure A3 (Appendix). (**B**) Quantification of DNA-PKcs protein levels compared to loading control and normalized to untreated (Ctrl). Averages and SEM are indicated. (**C**) PC346C-Flu1 cells were transfected with a pool of siRNAs against the DNA-PKcs or non-targeting siRNA for 48 h, and then treated with 2 Gy of IR treatment. Representative images of γH2AX and 53BP1 stainings 24 h post-IR (scale bar = 10 μm). (**D**) Quantification of γH2AX and 53BP1 foci. Each data point represents one cell; averages and SEM are indicated. (**E**) Representative pulsed-field gel electrophoresis (PFGE) image of PC346C-Flu1 cells treated as in C, followed by treatment with 15 Gy of IR. (**F**) DNA break signals were quantified by ImageJ and normalized to unsaturated signals of intact DNA. Averages and SD are indicated. (**G**) PC346C-Flu1 cells were treated as in C, followed by 1 Gy of IR treatment, and cells were counted seven days after treatment by sulforhodamine beta (SRB) assay with cell number set relative to initial cell number; * *p* < 0.05, *** *p* < 0.001, ns, non-significant. ADT, androgen-deprivation treatment; APA, apalutamide; ENZA, enzalutamide.

After excluding the HR pathway as the direct cause of AR suppression induced radiosensitization, we investigated the involvement of the NHEJ pathway. Previously, Goodwin et al. showed that the AR pathway regulates the NHEJ factor DNA-PKcs [7]. Therefore, we analyzed DNA-PKcs protein expression after AR suppression treatment. A significant reduction of the DNA-PKcs protein level was observed in all three cell lines (Figure 5A,B). On average, all AR suppression modalities reduced DNA-PKcs levels to 30–60%. We further investigated whether these reduced DNA-PKcs levels were sufficient to explain the observed DSB repair defects (Figure 3). To this end, PC346C-Flu1 cells were treated with small interfering RNA (siRNA) to reduce DNA-PKcs expression. This cell line was selected since HR was shown to be unaffected, allowing us to determine if reduction of NHEJ repair could explain the observed synergistic therapy response. DNA-PKcs expression was reduced to approximately 50% by siRNA treatment, similar to DNA-PKcs levels observed after AR suppression. A significantly decreased ability to repair DSBs was observed at 24 h after IR compared to control cells (Figure 5C,D). DNA-PKcs knockdown did show a significant difference in γH2AX and 53BP1 foci levels at 1 h post-IR as was observed for AR suppression treatments. Furthermore, PFGE analysis also showed a higher level of DNA breaks at 1 h after IR in cells with reduced DNA-PKcs levels compared to control cells (Figure 5E,F). The functional consequences of DNA-PKcs reduction were directly assessed, and these cells showed a significant reduced cell number after IR treatment compared to cells with normal levels of DNA-PKcs (Figure 5G). These results indicate that reduced DNA-PKcs expression caused by AR suppression treatment can at least partially account for the observed delay in DSB repair and the associated radiosensitization.

## 3. Discussion

Here, we demonstrate that different forms of AR suppression treatment can act as a radiosensitizer in AR-expressing androgen-dependent and CRPC preclinical PCa models. The mechanism of action causing this radiosensitization involves regulation of the NHEJ DNA repair pathway.

We and others found that AR suppression and IR treatment have an additive effect on cell proliferation in AR-expressing PCa [7,13]. Based on these data, AR suppression treatments were reported to have a radiosensitizing effect. However, the observed effects could also be explained by the additivity of both modalities. Previous reports came to opposing conclusions regarding the radiosensitizing effect of AR suppression in CRPC models, with studies showing a synergistic effect [7,15] or no effect [13,16]. Our in vitro CRPC cells and ex vivo androgen-dependent PCa tissue slices experiments provide new evidence that AR suppression can act as radiosensitizer in both AR-expressing hormone-sensitive PCa (which represents the majority of patients receiving EBRT) and AR-expressing CRPC. Although no synergy could be demonstrated in cell growth assays, our DSB repair kinetic assays showed a clear synergy between IR and AR suppression treatment for AR-expressing PCa cells. This result could not be confirmed by standard clonogenic survival assays since all our AR-expressing PCa cell lines had extremely low plating efficiency with very poor colony formation ability. We found similar effects of apalutamide, enzalutamide, and ADT, showing that various methods of AR suppression have similar efficacy in vitro. We conclude that patients with AR-expressing PCa can benefit from apalutamide or other AR suppressing treatment in addition to EBRT.

Previous preclinical studies investigated the molecular mechanism of radiosensitization by ADT and discovered an interplay between AR signaling and the DNA repair machinery [7,8,10,11,16]. However, opposing conclusions were drawn from these studies about the DNA repair pathways that cause the radiosensitization. Studies reported a functional link between AR signaling and HR repair and synthetic lethality in combinations of AR inhibitors and poly (ADP-ribose) polymerase (PARP) inhibitors in PCa. Enzalutamide suppressed the expression of the HR protein RAD51 in androgen-dependent PCa cells [10] and HR efficiency, measured by a functional HR assay, which was indeed significantly reduced by AR suppression [10,11,16]. We found that reduced RAD51 expression and compromised HR could be explained by a reduced fraction of S/G2 phase cells. Such cell-cycle analyses were not included in their studies, which precludes definitive conclusions. Asim et al. showed that IR-induced RAD51 foci formation in Ki67-positive PCa cells was strongly decreased in patient biopsies who received ADT [11]. This is seemingly in contrast with our results which show that equal numbers of RAD51 foci were detected in EdU-positive cells after AR suppression. However, this discrepancy can be explained by the difference in detection of proliferating cells; EdU labels only S-phase cells, whereas Ki67 is present in all cycling cells. It was even reported to persist for several days after cells ceased to proliferate [17]. Ki67-positive cells may, thus, be primarily G1-phase cells that neither form RAD51 foci nor perform HR. Together, our data indicate that the HR pathway is not directly regulated by AR signaling, but HR might be reduced on a cell population level caused by a change of the cell-cycle profile.

After excluding AR-regulated HR repair, we further investigated the NHEJ repair pathway. A previous study discovered that AR directly regulates the level and function of the NHEJ factor DNA-PKcs [7]. Our data confirmed this observation and showed that AR suppression reduced DNA-PKcs levels in both androgen-dependent and CRPC cells. Downregulation of DNA-PKcs is further supported by the low staining intensity of DNA-PKcs in PCa tissue after combined castration and radiotherapy when compared with radiotherapy alone [18]. These data were further confirmed by siRNA knockdown of DNA-PKcs indeed resulting in a similar level of radiosensitization and delaying DNA repair in AR-expressing cells. We conclude that NHEJ is directly regulated by AR signaling and that downregulation of DNA-PKcs expression contributes to the radiosensitization induced by AR suppression.

Previous preclinical studies reported the synthetic lethality between AR inhibitors and PARP inhibitors [10,11]. Unfortunately, the first randomized multicenter clinical trial failed to show a significant difference in prostate-specific antigen response rate and median progression-free survival between patients treated with abiraterone plus the PARP inhibitor veliparib and those treated with abiraterone acetate plus prednisone alone [19]. This combination was based on the rationale that tumors with loss-of-function mutations in BRCA1/2 are deficient in HR repair, which makes them highly sensitive to PARP inhibition treatment [20,21]. Based on our finding that AR suppression causes reduced NHEJ, not HR, one would indeed expect a lack of efficacy from this combination treatment in an unselected PCa patient population. On the other hand, PCa patients harboring BRCA1/2 mutations could possibly benefit to a greater degree from combination of apalutamide and EBRT compared to BRCA1/2 wild-type patients, as intrinsic HR repair defects in these patients plus compromised NHEJ repair by apalutamide would render the tumor cells exquisitely vulnerable to IR.

Emerging preclinial and clinical studies show that AR variants (AR-Vs) can play an important role in the development of resistance to AR suppression treatment [22,23]. AR-Vs lack the ligand-binding domain and, therefore, are constitutively active, which consitutes a mechanism of resistance when androgen ligands are very low under ADT [22]. Recently, studies reported that, in genetically engineered R1-D567 cells (only expressing AR-Vs) and the 22Rv1 cell line (expressing both AR and high AR-V7), combination of IR and AR suppression treatment could not increase radiosensitivity via altering the AR-mediated DNA damage response (DDR) [24]. These studies imply that the expression of AR-Vs in PCa cells may counteract the AR suppression-induced radiosensitization, and that patients with AR-V expression might not be eligible for such a combination regimen. It remains to be investigated whether a certain level of AR expression is a prerequisite for achieving radiosensitization by AR suppression treatments. These issues warrant further investigation with more PCa models.

We did not find significant differences in response between ADT, enzalutamide, and apalutamide treatment, probably because ADT in an in vitro setting is a simplified and optimal scenario, while effects in patients are less optimal due to adrenal androgen production being unaffected under ADT. Apalutamide recently received Food and Drug Administration (FDA) approval for the treatment of non-metastatic CRPC. Several clinical trials are underway to test the efficacy of apalutamide with EBRT (NCT03488810, NCT03503344, NCT02531516). The preclinical data presented here fully support the ongoing clinical trials. Furthermore, our study showed benefit of the combination in AR-expressing CRPC cells, a situation also found in patients with advanced metastatic disease that are progressive on ADT. Therefore, we propose that an alternative treatment approach should be considered for these patients, consisting of apalutamide in combination with prostate-specific membrane antigen (PSMA)-targeted radioligand therapy [25]. The decreased DNA repair effectivity in the tumor, but not the normal tissue, should increase the therapeutic ratio in this setting.

## 4. Materials and Methods

### 4.1. Reagents

Reagents were purchased from Sigma-Aldrich (Darmstadt, Germany), unless otherwise specified. The following antibodies were used: AR (1:200, M4074, SPRING Bioscience, Pleasanton, CA, USA), RAD51 (1:10000, homemade [26]), DNA-PKcs (1:1000, homemade [27]), phospho-histone H2AX (ser139) (γ-H2AX) (1:500, JBW301, Millipore, Darmstadt, Germany), 53BP1 (1:1000, NB100-904, Novus Biologicals, Littleton, CO, USA), α-tubulin (1:10,000, B-5-12, Sigma-Aldrich, Darmstadt, Germany), and anti-rabbit/mouse Alexa Fluor 488/594 (1:1000, Life Technologies, Carlsbad, CA, USA). Apalutamide was a gift from Janssen-Cilag B.V., and enzalutamide was purchased from Axon Medchem (Groningen, The Netherlands). Both compounds were diluted in dimethyl sulfoxide (DMSO) and used at a final concentration of 1µM.

### 4.2. Cell Lines

DU145 and LNCaP cells were purchased from American Type Culture Collection (Manassas, VA, USA) and cultured as described [28,29]. PC346C, PC346C-DCC, and PC346C-Flu1 cells were established in our laboratory and cultured as previously described [30]. Composition of the cell culture medium and characteristics of each cell line can be found in Table A1 (Appendix). All cells lines were authenticated for AR expression by Western blot. Cells were regularly tested for mycoplasma infection, and kept in culture for a maximum of 25 passages after initiating the hormone sensitive/castration resistance phenotype.

### 4.3. Irradiation (IR)

Cells and tissue slices were irradiated with different X-ray doses using Xstrahl Cabinet Irradiator RS320 (195 kV, 10 mA, 1.67 Gy/min, Xstrahl Ltd. Camberley, UK).

### 4.4. Cell Growth Assay

Cell growth was measured using the sulforhodamine beta (SRB) assay. Briefly, all cells were plated in 96-well plates coated with FNC coating mix (Athena Environmental Sciences) in 100 μL of steroid-stripped medium (medium with dextran-coated charcoal-treated FCS (DCC)). The next day, 100 µL of culture medium was added either alone (ADT condition) or supplemented with R1881 (0.1 nM, control condition) or R1881 (0.1 nM) plus apalutamide/enzalutamide (1 µM, apalutamide or enzalutamide condition) 24 h before irradiation (IR). Cells were allowed to grow until different time points, and fixed and stained with SRB as previously described [31]. Absorbance was measured at 560 nm using a GloMax^®^-Multi Detection System (Promega, Madison, WI, USA).

### 4.5. Immunofluorescent Staining on Cells

The γ-H2AX and 53BP1 staining was performed as previously described [31]. Briefly, cells were seeded in DCC medium overnight on coverslips and treated with various anti-androgen or control (R1881) treatments for 24 h before IR. Cells were fixed with 2% paraformaldehyde (PFA) after 1 h and 24 h. Following permeabilization and blocking, cells were incubated with anti-γH2AX and anti-53BP1 antibodies overnight, as well as secondary antibodies. EdU/RAD51 co-staining was done as previously reported [32]. Briefly, 5-ethynyl-2’-deoxyuridine (EdU, Thermo Fisher Scientific, Carlsbad, CA, USA) at a concentration of 3 μg/mL was added to the cells 30 min before fixation. Pre-extraction was performed in Triton X-100 buffer (0.5% Triton X-100, 20 mM 4-(2-hydroxyethyl)-1-piperazineethanesulfonic acid (Hepes)-KOH, pH 7.9, 50 mM NaCl, 3 mM MgCl_2_, 300 mM sucrose) at room temperature (RT) for 1 min before fixation. After permeabilization and blocking, cells were incubated with anti-RAD51 antibody and subsequently incubated with secondary antibody followed by Click-iT Alexa Fluor 594 cocktail buffer for 30 min.

### 4.6. Cell-Cycle Analysis

Cell-cycle distribution was measured by flowcytometry [33]. Briefly, cells were seeded in DCC medium overnight and treated with various antiandrogen treatments for 24 h before IR. EdU at a concentration of 3 μg/mL was added to the culture medium 30 min before fixation, and cells were fixed with ice-cold 70% ethanol 24 h post-IR. Cells were washed with phosphate-buffered saline (PBS) and permeabilized in 0.1% Triton X-100 in PBS for 5 min at RT. Subsequently, cells were incubated in Click-iT Alexa Fluor 594 cocktail buffer for 30 min, and then cells were resuspended in 1 μg/mL 4′,6-diamidino-2-phenylindole (DAPI) and 0.1 mg/mL RNase (Roche Life Sciences, Mannheim, Germany) in PBS. Cell-cycle distribution was measured using an LSRFORTESSA FACS machine (BD Bioscience, San Jose, CA, USA).

### 4.7. Tissue Slice Culture 

All animal experiments were approved by the Animal Welfare Committee of the Erasmus MC, and all experiments were conducted in accordance with accepted guidelines (permit AVD101002017867, 25 September 2017). PC295 PDXs were previously established in our laboratory [34]. PDX tumors were excised, sliced, and cultured as previously reported [14]. Briefly, 300-μm slices were cultured in advanced Dulbecco′s Modified Eagle′s Medium/F12 (ThermoFisher Scientific, Carlsbad, CA, USA) medium in six-well plates on a Rocking Table (Luckham 200 Ltd., West Sussex, UK) and one-third of the medium was refreshed daily. Slices were pretreated with apalutamide or DMSO control for 2 h and subsequently irradiated with a dose of 2 Gy. At different time points, slices were harvested and fixed in 10% neutral buffered formalin for 24 h at RT and embedded in paraffin; then, 4-μm sections were made for further analysis. EdU (3 μg/mL) was added to the tissue slice culture medium 2 h before fixation.

### 4.8. Tissue Section Terminal Deoxynucleotidyl Transferase dUTP Nick end Labeling (TUNEL) and EdU Click-IT Assays

EdU at a concentration of 3 μg/mL was added to the tissue slice culture medium 2 h before fixation. Simultaneous TUNEL and EdU staining was performed as described previously [35]. Briefly, tissue sections were deparaffinized in xylene followed by rehydration in graded alcohols and then blocked with PBS 3% bovine serum albumin (BSA). TUNEL reaction was performed using an In Situ Cell Death Detection Kit (Roche Life Sciences, Penzberg, Germany), after which the sections were incubated with Click-iT Alexa Fluor 594 (Invitrogen, Carlsbad, CA, USA) cocktail buffer for 30 min.

### 4.9. Tissue Section Hematoxylin and Eosin (H&E)

Histological tumor architecture was examined by H&E staining as previously described [14]. Briefly, sections were deparaffinized in xylene followed by rehydration in graded alcohols and staining with hematoxylin and eosin [14].

### 4.10. Tissue Section Immunohistochemical Staining

Sections were deparaffinized in xylene followed by rehydration in graded alcohols. Antigen retrieval was performed with target retrieval buffer (pH 6, Dako, Glostrup, Denmark). Sections were treated with 3% hydrogen peroxide solution in methanol at RT for 20 min, followed by incubation in 5% BSA in PBS for 30 min at RT. Sections were incubated with anti-AR antibodies overnight, and horseradish peroxidase (HRP)-conjugated anti-rabbit immunoglobulin G (IgG) secondary antibody (1:100, Dako) for 1 h at RT. AR-positive cells were visualized using a diaminobenzidine (DAB) staining kit (Agilent, Santa Clara, CA, USA) and hematoxylin counter-staining.

### 4.11. Pulsed-Field Gel Electrophoresis

Pulsed-field gel electrophoresis (PFGE) was performed as previously described [36]. Briefly, cells were embedded in 2% agarose plugs and lysed following 15 Gy of IR (15 Gy is commonly used for the PFGE assay since 2 Gy is too low to detect clear bands of DSBs). Electrophoresis was performed at 14 °C in 0.9% pulse-field certified agarose (Bio-Rad, Hercules, CA, USA) in a Bio-Rad Chef DR III system (Bio-Rad, La Jolla, CA, USA). The gel was stained with ethidium bromide and imaged on a UVITEC gel documentation system (UVItec, Cambridge, UK). DSBs bands were quantified using ImageJ (version 1.52, open source software via https://imagej.nih.gov/ij/index.html) with background subtracted and normalized to unsaturated signals of intact DNA. For each treatment, these values were normalized against their respective untreated controls to obtain the fold-change in DSBs.

### 4.12. HR Assay

The DR-GFP assay was performed to measure HR as previous described [33]. Briefly, cells were transfected with 1.5 μg of DR-GFP plasmid and an I-SceI expression vector or empty vector according to the manufacturer’s protocol (FuGENE HD (Promega) for LNCaP and Lipofectamine 3000 (Invitrogen) for PC346C-Flu1). Transfected cells were cultured in medium containing apalutamide or DMSO control for 48 h. BRCA1 knockdown was used as a positive control. GFP expression was analyzed by flow cytometry (LSRFORTESSA, BD Bioscience, San Jose, CA, USA).

### 4.13. Western Blot

Cells were lysed and lysates were resolved by sodium dodecyl sulfate polyacrylamide gel electrophoresis (SDS-PAGE) (Bio-Rad, Hercules, CA, USA), transferred to polyvinylidene difluoride membranes, and analyzed using antibodies described above. Quantification was conducted using ImageJ software.

### 4.14. RNA Interference

PC346C-Flu1 cells were transfected with either control or DNA-PKcs siRNA (Dharmacon, Lafayette, CO, USA) using Lipofectamine RNAi MAX (Life Technologies, Carlsbad, CA, USA). Transfected cells were cultured for 48 h and then treated as specified.

### 4.15. Image Acquisition and Quantification

For 53BP1, γH2AX, and RAD51 foci quantification, at least three random fields of view of Z-stack images were captured with a LSM700 confocal microscope (Zeiss, Oberkochen, Germany) and quantified as previously described [32]. AR and H&E was imaged with a light microscope (Olympus, Tokyo, Japan), and four fields of view from each section were analyzed. For EdU and TUNEL quantification, 10 random images from each tissue slice section were generated using a Leica fluorescence microscope (DM4000b, Wetzlar, Germany) and quantified using Otsu’s algorithm in a Matlab-based software as previous reported [14]. AR expression was quantified using ImageJ software as previous described [14].

### 4.16. Statistical Analysis

Experiments were performed in duplicate (foci kinetics) or triplicate (all other experiments). One-way ANOVA was used to compare more than two groups, and an unpaired Student’s *t*-test was used to analyze the differences between two groups. Statistical analysis and generation of graphs was performed using GraphPad Prism 6.0 (GraphPad, La Jolla, CA, USA).

## 5. Conclusions

In summary, we demonstrate for the first time that the next-generation AR inhibitor apalutamide acts as a radiosensitizer in AR-expressing androgen-dependent PCa and CRPC models. This radiosensitization is caused by inhibition of DNA repair by NHEJ, not HR. Our results suggest that apalutamide can not only be used in combination with EBRT for the treatment of androgen-dependent localized PCa, but also for AR-expressing CRPC patients.

## Figures and Tables

**Figure A3:**
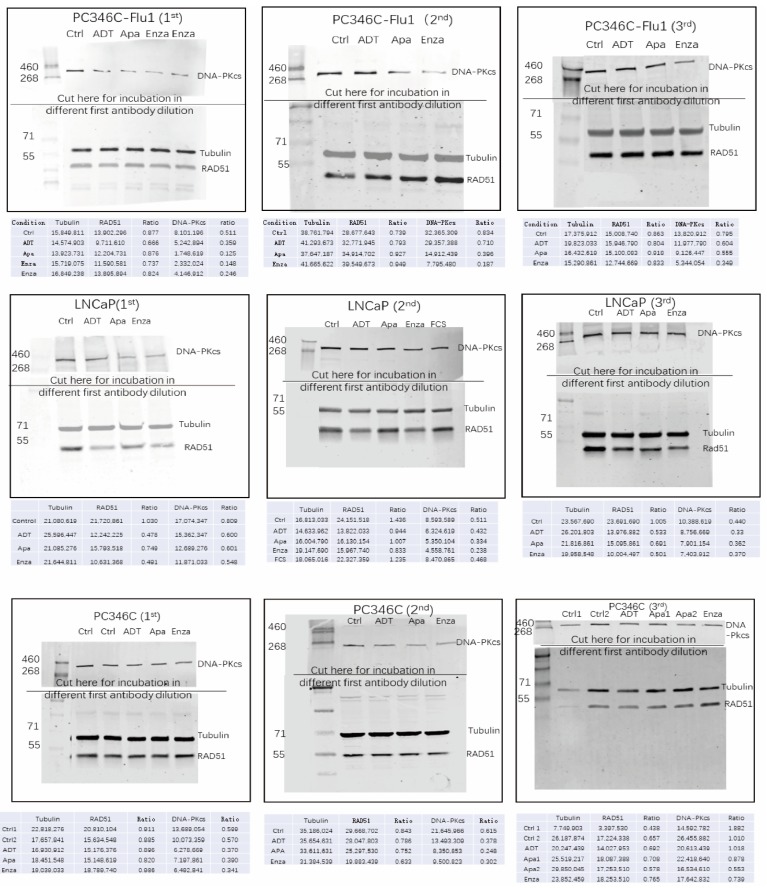
Full blots and quantification of the bands of the Western blot experiments shown in Figure 4 and Figure 5.

## Appendix A

**Table A1:** Characteristics of the used cell lines and composition of the cell culture media used for each cell line.

Cell Lines	DU145	LNCaP	PC346C	PC346C-DCC	PC346C-Flu1
**Culture** **Medium**	RPMI 1640 FCS (10%) PS	RPMI 1640 FCS (10%) PS	DMEM/Ham’s F12 BSA (0.01%) FCS (2%) Epidermal growth factor (10 ng/mL) Insulin–transferrin–selenium (1%) Hydrocortisone (0.5 µg/mL) Triiodothyronine (1 nM) Phosphoethanolamine (0.1 mM) Cholera toxin (50 ng/mL) Fibronectin (100 ng/mL) Fetuine (20 µg/mL) R1881 (0.1 nM) PS	DMEM/Ham’s F12 BSA (0.01%) DCC (2%) Epidermal growth factor (10 ng/mL) Insulin–transferrin–selenium (1%) Hydrocortisone (0.5 µg/mL) Triiodothyronine (1 nM) Phosphoethanolamine (0.1 mM) Cholera toxin (50 ng/mL) Fibronectin (100 ng/mL) Fetuine (20 µg/mL) R1881 (0.1 nM) PS	PC346C-DCC medium plus OH-flutamide (1 µM)
**AR**	–	+	+	−	++
**p53**	Missense Mutation	Silent	WT	WT	WT
**BRCA2**	Missense Mutation	WT	WT	WT	WT
**PTEN**	WT	Frame shift deletion	Nonsense mutation	Nonsense mutation	Nonsense mutation

RPMI: Roswell Park Memorial Institute; DMEM: Dulbecco′s Modified Eagle′s Medium; BSA: Bovine serum albumin; AR: Androgen receptor; BRCA2: Breast cancer 2; PTEN: Phosphatase and tensin homolog; PS: Penicillin/streptomycin (100 U/mL, 100 µg/mL); FCS: Fetal calf serum; DCC: Charcoal dextran-stripped serum; WT: Wild type. For DU145 and LNCaP, data were acquired from The Cancer Cell Line Encyclopedia (CCLE) https://portals.broadinstitute.org/ccle.
